# Immuno-PET Imaging of Atherosclerotic Plaques with [^89^Zr]Zr-Anti-CD40 mAb—Proof of Concept

**DOI:** 10.3390/biology11030408

**Published:** 2022-03-06

**Authors:** Kikkie Poels, Maxime Schreurs, Matthijs Jansen, Danielle J. Vugts, Tom T. P. Seijkens, Guus A. M. S. van Dongen, Esther Lutgens, Wissam Beaino

**Affiliations:** 1 Department of Medical Biochemistry, Amsterdam Cardiovascular Sciences (ACS), Amsterdam UMC, University of Amsterdam, 1105 AZ Amsterdam, The Netherlands; kikkie.poels@cshs.org (K.P.); matthijsfjansen@gmail.com (M.J.); t.t.seijkens@amsterdamumc.nl (T.T.P.S.); lutgens.esther@mayo.edu (E.L.); 2 Department of Radiology and Nuclear Medicine, Amsterdam UMC, Vrije Unversiteit, 1081 HV Amsterdam, The Netherlands; maximeschreurs5@hotmail.com (M.S.); d.vugts@amsterdamumc.nl (D.J.V.); gams.vandongen@amsterdamumc.nl (G.A.M.S.v.D.); 3 Department of Medical Oncology, Antoni van Leeuwenhoek–Netherlands Cancer Institute, 1066 CX Amsterdam, The Netherlands; 4 Institute for Cardiovascular Prevention (IPEK), Ludwig Maximilian’s University, 80336 Munich, Germany; 5 German Centre for Cardiovascular Research (DZHK), Partner Site Munich Heart Alliance, 80802 Munich, Germany

**Keywords:** CD40 imaging, atherosclerotic plaques, ^89^Zr-immuno-PET, PET-CT, cardiovascular disease, ex vivo biodistribution, macrophages

## Abstract

**Simple Summary:**

Atherosclerosis is characterized by gradual plaque build-up in the middle and large arteries and is the major cause of cardiovascular disease. Determining which plaques are prone to rupture and cause potential lethal effects (e.g., myocardial infarction) could greatly reduce the potential bad outcomes. CD40 is a co-stimulatory molecule present in various cells in the plaque and has been shown to correlate with plaque vulnerability. In this manuscript, we have combined a murine monoclonal antibody against CD40 (a costimulatory molecule directly linked to plaque progression and present on many cells within the lesion) with Zirconium-89 to test its applicability to detect lesions in a mouse model of atherosclerosis using PET/CT. We show that this Zirconium-89 labeled antibody can detect CD40 in atherosclerotic lesions in a mouse model of atherosclerosis. In wild type mice without plaques, no signal was found. Our results suggest that CD40 could be a potential marker for PET imaging of plaque inflammation and vulnerability.

**Abstract:**

Non-invasive imaging of atherosclerosis can help in the identification of vulnerable plaque lesions. CD40 is a co-stimulatory molecule present on various immune and non-immune cells in the plaques and is linked to inflammation and plaque instability. We hypothesize that a ^89^Zr-labeled anti-CD40 monoclonal antibody (mAb) tracer has the potential to bind to cells present in atherosclerotic lesions and that CD40 Positron Emission Tomography (PET) can contribute to the detection of vulnerable atherosclerotic plaque lesions. To study this, wild-type (WT) and ApoE^−/−^ mice were fed a high cholesterol diet for 14 weeks to develop atherosclerosis. Mice were injected with [^89^Zr]Zr-anti-CD40 mAb and the aortic uptake was evaluated and quantified using PET/Computed Tomography (CT) imaging. Ex vivo biodistribution was performed post-PET imaging and the uptake in the aorta was assessed with autoradiography and compared with Oil red O staining to determine the tracer potential to detect atherosclerotic plaques. On day 3 and 7 post injection, analysis of [^89^Zr]Zr-anti-CD40 mAb PET/CT scans showed a more pronounced aortic signal in ApoE^−/−^ compared to WT mice with an increased aorta-to-blood uptake ratio. Autoradiography revealed [^89^Zr]Zr-anti-CD40 mAb uptake in atherosclerotic plaque areas in ApoE^−/−^ mice, while no signal was found in WT mice. Clear overlap was observed between plaque areas as identified by Oil red O staining and autoradiography signal of [^89^Zr]Zr-anti-CD40 mAb in ApoE^−/−^ mice. In this proof of concept study, we showed that PET/CT with [^89^Zr]Zr-anti-CD40 mAb can detect atherosclerotic plaques. As CD40 is associated with plaque vulnerability, [^89^Zr]Zr-anti-CD40 mAb has the potential to become a tracer to detect vulnerable atherosclerotic plaques.

## 1. Introduction

Atherosclerosis is a chronic inflammatory disease of the middle- and large-sized arteries that results in the establishment of plaques in the arterial wall. While atherosclerosis often starts from an early age, its detrimental effects do not become apparent until plaques become advanced and lead to potentially lethal consequences such as myocardial infarction (MI) and ischemic stroke [1]. In 2016, 84.9% of cardiovascular deaths were caused by MIs and strokes, both the result of atherosclerosis, amounting to 15.1 million deaths worldwide per year [2]. Early detection and stabilization of unstable plaques could potentially eliminate these lethal ramifications and reduce cardiovascular death. Nevertheless, many factors contribute to a vulnerable plaque phenotype, and while advancements have been made in the latest years, it is still unclear what is the determining factor that ultimately drives plaques to rupture and cause cardiovascular complications [3]. Interestingly, plaques with a vulnerable phenotype still have the potential for stabilization, either spontaneously or drug-induced [4,5], so their identification has major therapeutic impact. For example, in the YELLOW trial, intensive treatment with statins significantly reduced the lipid core of advanced lesions and decreased the fractional flow reserve, indicating clinical improvement in patients with coronary artery disease [6]. Additionally, intra-vital ultrasound (IVUS) in swine showed that spontaneous remodeling was present in almost all advanced carotid lesions and extensive follow-up indicated that the most progressed plaques all reduced in volume after remodeling [5]. While remodeling and restabilization of plaques greatly decreases the chance of rupture of inflammatory plaques, patients with such restabilized lesions are often at increased risk for developing vulnerable lesions elsewhere in the vasculature and are still at risk for developing MI [7].

Current imaging strategies are able to detect many facets of plaque vulnerability, including inflammation ([^18^F]FDG PET), microcalcifications ([^18^F]NaF PET), necrotic cores (Computed Tomography (CT), IVUS), and thin fibrous caps (Optical Coherence Tomography (OCT)) [7]. Many of these imaging tools have some predictive value for identifying vulnerable lesions when used individually, but often overestimate at-risk plaques. For example, in the PROSPECT trial, 596 thin-cap atheroma were detected via IVUS, but after 3.4 years only 6 patients had suffered from MI [8]. It is thus important not to use only one plaque characteristic to predict cardiovascular risk, but to find the ultimate combination of contributing factors which could become a reliable predictor of plaque rupture. This was exemplified in the CLIMA study, where patients underwent OCT of the left anterior descending coronary artery and were followed for twelve months [9]. The combined presence of four risk factors in a single plaque (minimum lumen area < 3.5 mm^2^, fibrous cap thickness < 0.75 mm^2^, macrophage presence, and lipid arc circumferential extension > 180°) was associated with a 7-fold increase in the composite primary endpoint (including cardiac death and (non-fatal) MI) [9]. While this finding has greatly improved the predictive value of OCT scans, this combinational strategy does not take any inflammatory aspects besides macrophage presence into account. Macrophages can have a plethora of phenotypes, ranging from pro-atherogenic M1 macrophages to more atheroprotective M2 macrophages [10]. Sole presence of macrophages does not distinguish between these distinctly different phenotypes. It could thus help to find a marker that can identify M1 macrophages in the plaque and combine that with other factors of plaque vulnerability to further improve the detection of unstable lesions.

CD40 is a co-stimulatory molecule that is present on a plethora of cell types, including B cells, dendritic cells, and macrophages. The interaction of CD40 with its ligand CD40L on T cells promotes T cell activation and is crucial for modulating inflammatory responses [11]. The role of CD40 in atherosclerosis has been extensively studied by our group and others. Both CD40 and CD40L are expressed in human atherosclerotic lesions [12], and showed increased expression when atherosclerosis progresses, with highest expression in vulnerable plaques. Inhibition of CD40L or CD40 signaling, either via genetic deficiency or antibody and small molecule mediated inhibition, strongly decreased atherosclerotic plaque burden and induced a stable atherosclerotic plaque phenotype, showing a direct effect of CD40 in inducing plaque vulnerability [12,13,14]. Therefore, visualizing CD40 by PET in atherosclerosis has the potential to predict plaque vulnerability. The benefit over the more conventional [^18^F]FDG PET lies in the more widespread expression of CD40 in the plaque and its correlation with plaque vulnerability. [^18^F]FDG is only able to detect highly glycolytic macrophages in the vessel wall [15], whereas CD40 is not only highly expressed on these activated macrophages, but is also present on activated endothelial cells, smooth muscle cells, and B cells, which all contribute to plaque vulnerability [16]. We therefore hypothesize that CD40 might be an excellent novel target for non-invasive imaging of atherosclerotic plaques. ^89^Zr is a radioactive isotope that can be bound to monoclonal antibodies. PET imaging with ^89^Zr-labeled antibodies is already used in cancer patients to identify tumor antigens for targeted treatment [17,18]. ^89^Zr has a half-life of 78.4 h, which allows PET imaging many days post-tracer administration, which is needed for optimal lesion to background ratios [19]. In this study, we radiolabeled an anti-CD40 monoclonal antibody (mAb) with ^89^Zr to explore its potential as a diagnostic tool for vulnerable plaques identification. We evaluated the uptake of [^89^Zr]Zr-anti-CD40 mAb in the ApoE^−/−^ atherosclerosis mouse model using PET imaging. We subsequently performed ex vivo biodistribution and autoradiography to validate plaque targeting.

## 2. Materials and Methods

### 2.1. Preparation of ^89^Zr-Labeled Anti-Mouse CD40 Antibody and Quality Controls

[^89^Zr]Zr-DFO*-NCS-anti-CD40 was produced based on the method described by Chomet et al. [20]. In short, 2.5 mg of the anti-CD40 antibody (anti-mouse CD40; BE0016-2; BioXCell, West Lebanon, NH, USA) were rebuffered into 0.9% NaCl using a size exclusion chromatography PD10 column (GE Healthcare, Eindhoven, The Netherlands), and the protein content of the fractions containing product was determined using nanodrop (280 nm, NanoVue Plus, GE Healthcare Life Sciences, Hoevelaken, The Netherlands). After buffer exchange, the mAb was concentrated by centrifugal ultrafiltration at 4000 rpm for 15 min using a spinfilter (Amicon^®^ Ultra-4 10K, Merck-Millipore, Burlington, MA, USA) and diluted to 0.5 mL with 0.9% NaCl. The pH of the mAb was adjusted to 8.9–9.1 with 0.1 M Na_2_CO_3_ and the mAb solution was added to 5 equivalents of DFO*-NCS (ABX, Radeberg, Germany) in DMSO (5 mM, 16.7 µL) and incubated for 120 min in a Thermomixer (550 rpm) at 37 °C. Hereafter, DFO*-NCS-anti-CD40 mAb was purified over a PD10 column (preconditioned and eluted with 0.9% NaCl) to remove non-conjugated hydrolyzed chelator. The concentration of DFO*-NCS-anti-CD40 mAb was determined using a calibration curve on size-exclusion high performance liquid chromatography (SE-HPLC). Radiolabeling of the DFO*-NCS-anti-CD40 mAb was performed in a 1 mL or 2 mL reaction volume. For the 1 mL reaction, the following protocol was executed: 100 µL 89Zr (~70 MBq, Perkin Elmer, Boston, USA) in 1 M oxalic acid and 45 µL of 2 M Na_2_CO_3_ were added together and reacted for 3 min. Subsequently, 250 µL of 1.0 M HEPES (pH 7.0) and 605 µL of DFO*-NCS-anti-CD40 mAb (±0.7 mg) were added. For the 2 mL reaction, all the volumes of the reagents were doubled. The solutions were put on a shaker for 60 min at room temperature. [^89^Zr]Zr-DFO*-NCS-anti-CD40 mAb (named [^89^Zr]Zr-anti-CD40 in the rest of the manuscript) was purified using prewashed PD10 columns and collected in 1.5–2.0 mL formulation buffer (20 mM sodium acetate + 200 mM sucrose + 0.01% Tween 20 pH 5.4–5.6). Hereafter, the concentration of the product was determined using a calibration curve on SE-HPLC). Finally, the product was formulated to 5 MBq-100 µg [^89^Zr]Zr-anti-CD40 mAb in 150 µL per mouse for the ApoE^−/−^ “low dose” and WT group and 5 MBq-1000 µg in 200 µL for the ApoE^−/−^ “high dose” group (see below).

### 2.2. Quality Controls

After labeling, the [^89^Zr]Zr-anti-CD40 mAb was checked for radiochemical purity, antibody integrity and immunoreactive fraction. Spinfilter analysis was performed to measure the radiochemical purity. Accordingly, 4 µL of product was added to 96 µL wash buffer (5% DMSO and 95% formulation buffer). The solution was applied to a filter unit (Micronon-30, Merck-Millipore, Amsterdam, The Netherlands) and centrifuged for 7 min at 14,000 rpm. Afterwards, the filter was washed two times with 100 µL of wash buffer and centrifuged (7 min, 14,000 rpm). After the last step, the filter containing the [^89^Zr]Zr-anti-CD40 mAb and the filtrate containing free ^89^Zr and unconjugated [^89^Zr]Zr-DFO*-NCS were measured with a gamma counter (Wallac LKB-CompuGamma 1282; Pharmacia, Stockholm, Sweden) for radioactivity content. The radiochemical purity was calculated by the ratio between the radioactivity content on the filter and the total radioactivity content (filter + filtrate).

The radiochemical purity, protein content, and antibody integrity were measured by SE-HPLC. To this end, a Shimadzu HPLC was equipped with a Superdex^®^ 200 Increase 10/300 GL (30 cm × 10 mm, 8.6 μm) size exclusion column (GE Healthcare Life Sciences, Hoevelaken, The Netherlands), including a guard column, using a solution of 0.05 M phosphate buffer/0.15 M NaCl/0.01 M NaN_3_ (pH 6.7) as eluent at a flow rate of 0.75 mL/min (total run time 40 min). Radioactivity was monitored with a radioactivity detector and defined as the percentage of the area under curve of the radiolabeled mAb compared to the total area on the radioactive channel. Antibody integrity was expressed as the percentage of the area under the curve on the UV 280 nm channel. The concentration was determined against a calibration curve of the parental mAb.

A binding assay was performed to assess the immunoreactive fraction of the radiolabeled construct binding to recombinant mouse CD40 (rmCD40, R&D Systems, Minneapolis, MN, USA) protein. rmCD40 was diluted in a coating buffer (15 mM sodium carbonate/35 mM sodium bicarbonate/3 mM sodium azide buffer, pH 9.3 to 9.8) to a concentration of 2 µg/mL and 100 µL was applied to MaxiSorp™ break-apart wells (Invitrogen™, Waltham, MA, USA) and incubated overnight at RT. The next day the excess rmCD40 was removed, and the wells were washed three times with PBS (150 µL), followed by blocking the plates with a 1% BSA/PBS solution for 60 min at room temperature while shaking. A serial dilution (ranging from 2 µg/mL to 31 ng/mL) of the radiolabeled product in 1% BSA/PBS was made in triplicate. The lowest concentration was tested twice, with and without addition of cold mAb (4 µL) to assess non-specific binding. After blocking, the plates were washed with a 0.05% Tween 20/ PBS solution (200 µL) and subsequently 100 µL of each diluted [^89^Zr]Zr-anti-CD40 mAb product was added to each coated well and incubated overnight at 4 °C. The next day, supernatant of each well was collected. The wells were washed twice with 0.05% Tween 20/PBS and the washing fractions were collected and pooled with the supernatant collected earlier. The wells and supernatants were counted in a gamma counter. The immunoreactive fraction was determined by percentage bound to rmCD40-coated wells compared to the total amount of radioactivity per sample.

### 2.3. Animals

Female ApoE^−/−^ mice (B6.129P2-Apoetm1Unc/J, 8 weeks old, Charles River, N = 12) were used for this study. This animal model contains a homozygous deletion of the ApoE gene resulting in a decreased clearance of lipoproteins (e.g., LDL) from the blood, which leads to the spontaneous development of atherosclerotic plaques [21]. To accelerate the development of the atherosclerotic plaques the mice were fed a high fat diet (0.15% cholesterol and 16% fat, Altromin, Lage, Germany) for 14 weeks. Age-matched wild-type (WT) female (C57BL/6J, Charles River, N = 6) mice fed a standard chow diet for an equal period of time served as controls. Mice were housed under standard laboratory conditions with water and food ad libitum.

Animal experiments were performed in accordance with the European Community Council Directive (2010/63/EU) for laboratory animal care and the Dutch Law on animal experimentation. The experimental protocol was validated and approved by the central committee for animal experimentation (CCD) and the local committee on animal experimentation of Amsterdam UMC, location VUmc (Study number: AVD1140020174049).

### 2.4. Study Design

To evaluate the potential of [^89^Zr]Zr-anti-CD40 mAb as PET tracer for detecting vulnerable plaques, ApoE^−/−^ mice fed a high fat diet for 14 weeks were intravenously injected with either [^89^Zr]Zr-anti-CD40 mAb (100 µg, 5 MBq in 150 µL, N = 6, “low dose”) or [^89^Zr]Zr-anti-CD40 mAb supplemented with additional cold mAb (1000 µg, 5 MBq in 200 µL, N = 6, “ high dose”). The two doses were compared to evaluate the effect of the dose on the blood kinetics of [^89^Zr]Zr-anti-CD40 mAb, level of uptake in the plaques, and lesion to background signal. WT mice were injected intravenously with [^89^Zr]Zr-anti-CD40 (100 µg, 5 MBq, N = 6), and referred herein as controls. PET scans followed by a contrast enhanced CT scan to visualize the blood vasculature were performed at day 3 and 7 p.i. PET quantification could not be performed in one animal in the “low dose” [^89^Zr]Zr-anti-CD40 group, in two animals in the “high dose” [^89^Zr]Zr-anti-CD40, and in two animals in the WT group due to failed contrast CT for vasculature visualization. In the “high dose” [^89^Zr]Zr-anti-CD40, one animal died during the experiment. At day 7, immediately after PET imaging, animals were sacrificed and perfused to remove the blood content. The aorta and its main branch points along with all other organs were carefully removed for assessment of ex vivo biodistribution and further analysis. For assessment of pharmacokinetics, blood samples were collected from the tail vein at days 1, 2, and 3 and by heart puncture at day 7 p.i.

### 2.5. PET/CT Acquisition and Quantification

PET images were acquired on a Mediso nanoScan PET/CT (Mediso Ltd., Budapest, Hungary). Mice were under isoflurane anesthesia (1.5–2.5%, 1 L/min oxygen) for the duration of the scan. Mice were positioned in the imaging chamber with integrated hot-air channels temperature control (37 °C). The respiratory rate was monitored during the entire scan via a breathing sensor. A computed tomography (CT) (5 min) was performed to acquire morphological data for image processing and reconstruction and identification of organs and tissues of interest. The CT was followed by a 60 min PET scan. At the end of each PET scan an additional CT scan (2 min) was acquired while injecting contrast agent (Iomeron 400, Bracco Diagnostics, Oxford, UK). Contrast agent was injected intravenously via tail vein catheter (250 μL, speed rate of 200 µL/min) using an intravenous pump, to visualize the cardiovascular structures (e.g., blood vessels).

PET image reconstruction was performed using a fully 3-dimensional reconstruction algorithm (Tera-Tomo^TM^, Mediso Ltd., Budapest, Hungary) with 4 iterations and 6 subsets, and an isotropic 0.4 mm voxel dimension. Scattering, and attenuation correction were applied.

Images were analyzed and quantified using VivoQuant software (version 4.0, Invicro, Boston, MA, USA). Regions of interest (ROIs) were drawn on the aorta arch, spleen, liver, muscle, and lymph nodes and tracer concentration were extracted from the PET images (Figure S1). Blood tracer content was determined by drawing a ROI on the left ventricle (LV). Radioactivity values were decay corrected to the injection time, and the radioactivity uptake was calculated as the percentage of the injected dose per gram of tissue (%ID/g). The aorta-to-blood ratio was calculated by dividing the %ID/g of the aorta arch with the %ID/g of total blood determined from the LV.

### 2.6. Ex Vivo Biodistribution

The ex vivo biodistribution was assessed at 7 days post injection after the last PET/CT scan. Mice were anesthetized, a blood sample was taken via heart puncture followed by perfusion with PBS. The aorta and its main branch points, along with all other organs were carefully removed for further analysis. The tissues were weighed, and the radioactivity in each of the organs was measured in a gamma counter (Wallac LKB-CompuGamma 1282; Pharmacia, Stockholm, Sweden). The radioactivity uptake was calculated as the percentage of the injected dose per gram of tissue (%ID/g).

### 2.7. Oil Red O Staining and Autoradiography

The aortic arch was dissected from the mice and then cut open in the longitudinal direction to enable the visualization of the plaques. Complete aortas and their branch points were stained with Oil Red O to identify lipid-rich plaque regions. For autoradiography, complete aortas were exposed to phosphor screen (BAS-IP SR 2040) for~1 week. After exposure, the screens were imaged using Typhoon FLA 7000 imager (GE Healthcare, Hoevelaken, The Netherlands) and the signal was quantified using Image Quant. ROIs were drawn on each individual plaque to determine the average intensity. For comparison between tissues exposed on different screens, the data were corrected for the difference in exposure time and the cross-comparison factor generated by standards.

### 2.8. Statistics

Data are represented as mean ± standard deviation (SD). Statistical comparisons were made using a non-parametric Mann–Whitney test. Data were analyzed using Graphpad prism 9 (San Diego, CA, USA). A *p*-value < 0.05 was considered significant. The Grubbs outlier test was used to check for outliers.

## 3. Results

### 3.1. Radiolabeling of [^89^Zr]Zr-Anti-CD40 mAb

The radiolabeling of anti-CD40 mAb with Zirconium-89 was achieved in a radiochemical yield of 67 ± 1% and a radiochemical purity of 99.2 ± 0.5% with a maximum binding of 80.6 ± 2.9% to rmCD40. The antibody integrity was 98.5 ± 0.2% according to the SEC-HPLC analysis. The corresponding chromatograms are shown in the Supplementary Material (Figures S2–S4).

### 3.2. Immuno-PET Imaging with [^89^Zr]Zr-Anti-CD40 mAb

**Figure 1:**
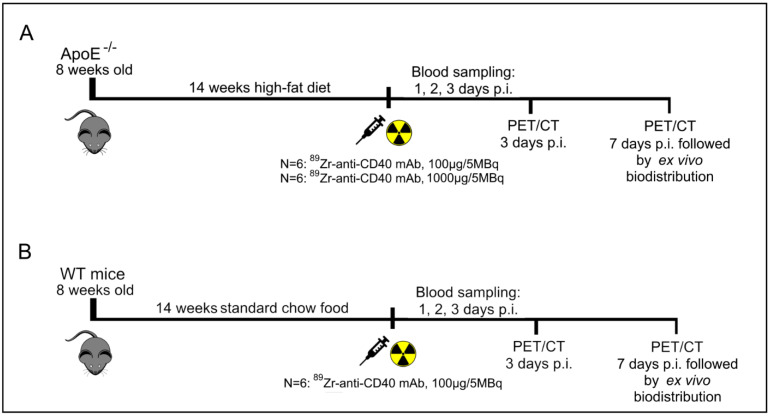
Schematic overview of the experimental design of PET and ex vivo biodistribution studies with [^89^Zr]Zr-anti-CD40 mAb. (**A**) Timeline for the ApoE^−/−^ mice injected with either a “low dose” of 100 µg-5 MBq (N = 6) or “high dose” of 1000 µg-5 MBq [^89^Zr]Zr-anti-CD40 mAb (N = 6). (**B**) The timeline for the WT mice injected with 100 µg-5 MBq [^89^Zr]Zr-anti-CD40 mAb (N = 6).

**Figure 2:**
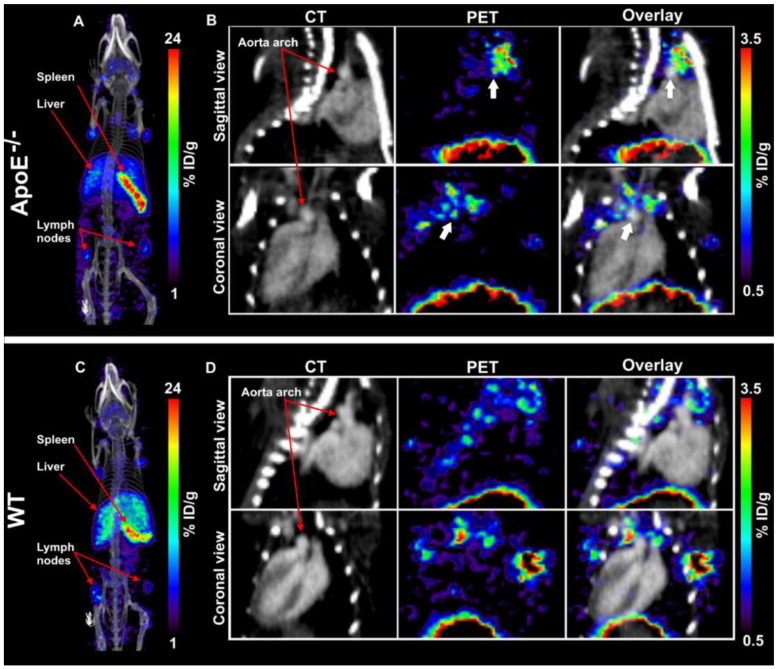
Representative PET/CT image of a ApoE^−/−^ and WT mouse 7 days after administration of [^89^Zr]Zr-anti-CD40 mAb. (**A**,**C**) MIP images of ApoE^−/−^ and WT mouse. (**B**,**D**) Tracer distribution in aorta of ApoE^−/−^ and WT mouse: CT image (**left**), aortic arch indicated by red arrows, PET image (**middle**), and CT image overlayed with PET (**right**). White arrows indicate apparent uptake in plaques. Data is expressed in percent injected dose per gram tissue (%ID/g).

A schematic overview of the study set-up can be found in Figure 1. In short, PET/CT imaging was performed at day 3 and 7 p.i. to evaluate the potential of [^89^Zr]Zr-anti-CD40 mAb (“low dose” of 100 µg) for in vivo targeting and detection of aortic plaques. Representative PET/CT images of [^89^Zr]Zr-anti-CD40 mAb in ApoE^−/−^ and WT mouse at day 7 p.i. are shown in Figure 2. The tracer mainly distributed to the liver and CD40-rich organs such as spleen and lymph nodes (Figure 2A,C). Additionally, focal uptake of [^89^Zr]Zr-anti-CD40 mAb was observed in the aortic arch of the ApoE^−/−^ mice (Figure 2B), which was not observed in the aortic arch of WT mice (Figure 2D), indicating binding to the atherosclerotic plaques that are present in the aorta of ApoE^−/−^ mice.

In order to obtain a better insight in the selective accumulation of the [^89^Zr]Zr-anti-CD40 mAb in the aortic arch of ApoE^−/−^ compared to the WT mice, we quantified the uptake in the aorta and organs from the PET images. The regions of interest were delineated using the CT morphological information as shown in Figure S1B.

**Figure 3:**
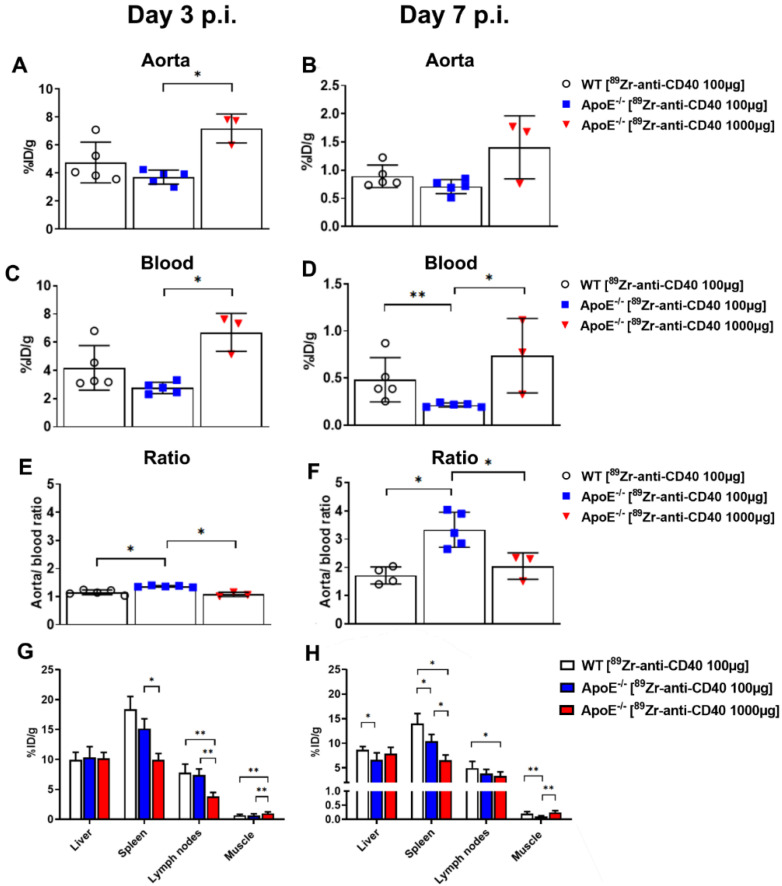
PET quantification of [^89^Zr]Zr-anti-CD40 mAb uptake in different organs of ApoE^−/−^ and WT mice at day 3 and 7 p.i. (**A**,**B**) Percent injected dose per gram tissue (%ID/g) in aorta. (**C**,**D**) %ID/g in blood (measured in left ventricle). (**E**,**F**) Aorta-to-blood ratio. (**G**,**H**) Tracer uptake in liver, spleen, lymph nodes and muscle, presented as percent injected dose per gram tissue (%ID/g). (* *p* < 0.05, ** *p* < 0.01; ApoE^−/− “^low dose”: N = 5 at day 3 and day 7 p.i., WT N = 4 at day 3 p.i. and N = 5 at day 7 p.i., and ApoE^−/−“^high dose” N = 3 at day 3 and day 7 p.i.).

At day 3 p.i., the uptake of [^89^Zr]Zr-anti-CD40 mAb “low dose” in the aortic arch was lower in the ApoE^−/−^ group (3.70 ± 0.50 %ID/g) compared to the WT group (4.74 ± 1.48 %ID/g; *p* = 0.31) (Figure 3A). However, the blood content showed the same trend (2.75 ± 0.40 vs. 4.18 ± 1.58 %ID/g, respectively) (Figure 3C, Table S1). To correct for the difference in blood content between the experimental groups, quantification of aortic arch uptake was performed by assessment of the aorta-to-blood ratio. This revealed significantly higher aorta-to-blood ratios in ApoE^−/−^ mice compared to WT animals (1.37 ± 0.03 vs. 1.16 ± 0.08, respectively; *p* < 0.05) (Figure 3E, Table S1). At day 7 p.i., a similar pattern was observed with higher aorta and blood uptake in the WT compared to the ApoE^−/−^ mice (Figure 3B,D, Table S2). However, the aorta-to-blood ratio was again significantly higher in the ApoE^−/−^ mice (3.34 ± 0.62) compared to WT mice (1.72 ± 0.30; *p* < 0.05) (Figure 3F, Table S2). A high uptake was also observed in the spleen and lymph node of ApoE^−/−^ mice at day 3 and 7 p.i., which is in accordance with the high level of CD40 expression in these organs. Liver uptake was comparable between WT and ApoE^−/−^ mice (Figure 3G,H, Tables S1 and S2).

High expression of CD40 in healthy organs might induce a sink effect that alters the antibody kinetics and atherosclerotic plaque targeting. In certain cases, a saturation of the sink organs can improve targeting to the organs of interest [22]. To investigate this, we evaluated the effect of a higher mass dose (“high dose” of 1000 µg) on the pharmacokinetics and aorta uptake of [^89^Zr]Zr-anti-CD40 mAb in ApoE^−/−^ mice.

Higher [^89^Zr]Zr-anti-CD40 mAb uptake was observed in the aorta with the “high dose” compared to the “low dose” at day 3 and 7 p.i. (6.73 ± 0.65 %ID/g vs. 3.70 ± 0.50 %ID/g; 1.41 ± 0.56 %ID/g vs. 0.71 ± 0.13 %ID/g; *p* < 0.05, respectively). Additionally, significantly higher [^89^Zr]Zr-anti-CD40 mAb levels were observed in the blood with the “high dose” of antibody (day 3: 6.70 ± 1.35 %ID/g vs. 2.75 ± 0.40 %ID/g; day 7: 0.74 ± 0.40 %ID/g vs. 0.21 ± 0.02 %ID/g; “high and low dose”, respectively) which indicate a CD40 sink effect. Aorta-to-blood ratios were significantly lower for the “high dose” compared to the “low dose” at day 3 (1.08 ± 0.07 vs. 1.37 ± 0.03, respectively) and day 7 (2.05 ± 0.47 vs. 3.34 ± 0.62, respectively) (Figure 3, Tables S1 and S2). Additionally, the aorta-to-blood ratio of the “high dose” in ApoE^−/−^ mice was comparable to WT mice dosed with 100 μg mAb. This indicates that a high mass dose of [^89^Zr]Zr-anti-CD40 mAb is not favorable for PET imaging of plaques due to the slower blood kinetics that leads to poor image contrast. In the CD40-rich organs, spleen, and lymph nodes, we observed lower uptake of [^89^Zr]Zr-anti-CD40 mAb in the “high dose” compared to the “low dose”, indicating a blocking effect in these organs and demonstrating the specificity of the tracer for targeting CD40 in vivo. Similar levels of [^89^Zr]Zr-anti-CD40 mAb uptake were observed in the liver for both doses (Figure 3G,H).

### 3.3. Ex Vivo Biodistribution and Blood Kinetics of [^89^Zr]Zr-Anti-CD40 mAb

**Figure 4:**
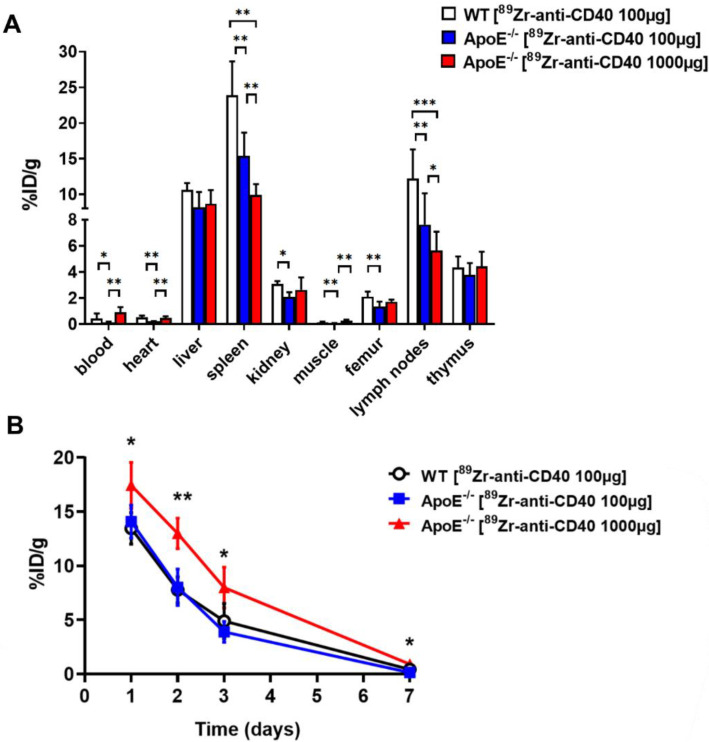
Ex vivo biodistribution results of [^89^Zr]Zr-anti-CD40 mAb. (**A**) Organ distribution at 7 days p.i. in ApoE^−/−^mice with “low dose” and “high dose”. (**B**) Blood kinetics curves in WT mice and ApoE^−/−^ mice with “low dose” and “high dose”. Uptake expressed as percent injected dose per gram tissue (%ID/g) (average ± SD) (* *p* < 0.05, ** *p* < 0.01, and *** *p* < 0.001; WT N = 6, ApoE^−/−^ “low dose” N = 6, and ApoE^−/−^ “high dose” N = 5).

To validate the PET imaging observations and results, we performed ex vivo biodistribution immediately after PET imaging acquisition at day 7. Blood samples were also collected at different time points to assess the blood kinetics of [^89^Zr]Zr-anti-CD40 mAb in WT and ApoE^−/−^ mice at “low dose” and “high dose”. As the aortas were too small for accurate quantification of ex vivo biodistribution, aortas were used for autoradiography analysis. Ex vivo biodistribution results showed high uptake of [^89^Zr]Zr-anti-CD40 mAb “low dose” in spleen (15.39 ± 3.26 %ID/g) and lymph nodes (7.62 ± 2.51 %ID/g) at day 7 p.i., which is in accordance with the PET imaging quantification (10.41 ± 1.38 and 3.88 ± 0.97 %ID/g, respectively). Additionally, the uptake of the tracer in spleen and lymph nodes was significantly lower when the mice were dosed with the “high dose” tracer (1000 µg) compared to the “low dose” tracer (100 µg) (spleen: 9.92 ± 1.52 %ID/g vs. 15.39 ± 3.26 %ID/g, *p* < 0.01 and lymph nodes: 5.66 ± 1.43 %ID/g vs. 7.62 ± 2.51 %ID/g; *p* < 0.05) (Figure 4A, Table S3). Liver and kidney uptake was comparable between the two doses, showing no effect of mAb dose on the catabolism and clearance of the tracer.

The [^89^Zr]Zr-anti-CD40 mAb showed similar blood kinetics in the WT and ApoE^−/−^ mice at the “low dose” at day 1 (13.47 ± 1.45 and 14.09 ± 1.52 %ID/g, respectively) and day 2 (7.78 ± 1.20 and 8.02 ± 1.68 %ID/g, respectively) post injection. At day 3 and 7 a faster washout of the tracer from the blood was observed in the ApoE^−/−^ compared the to the WT mice (day 3: 3.90 ± 0.95 vs. 4.90 ± 1.62 %ID/g; day 7: 0.15 ± 0.04 vs. 0.43 ± 0.39 %ID/g, respectively). However, significantly slower kinetics were observed when mice were injected with the “high dose” (Figure 4B, Table S4). At day 7 p.i., blood levels were significant higher in the ApoE^−/−^ “high dose” group compared to the ApoE^−/−^ “low dose” group (0.92 ± 0.38 vs. 0.15 ± 0.04 %ID/g, respectively; *p* < 0.01), but not significantly different from the control group (0.43 ± 0.39 %ID/g). These results are in accordance with the PET imaging quantifications.

### 3.4. [^89^Zr]Zr-Anti-CD40 mAb Ex Vivo Autoradiography

**Figure 5:**
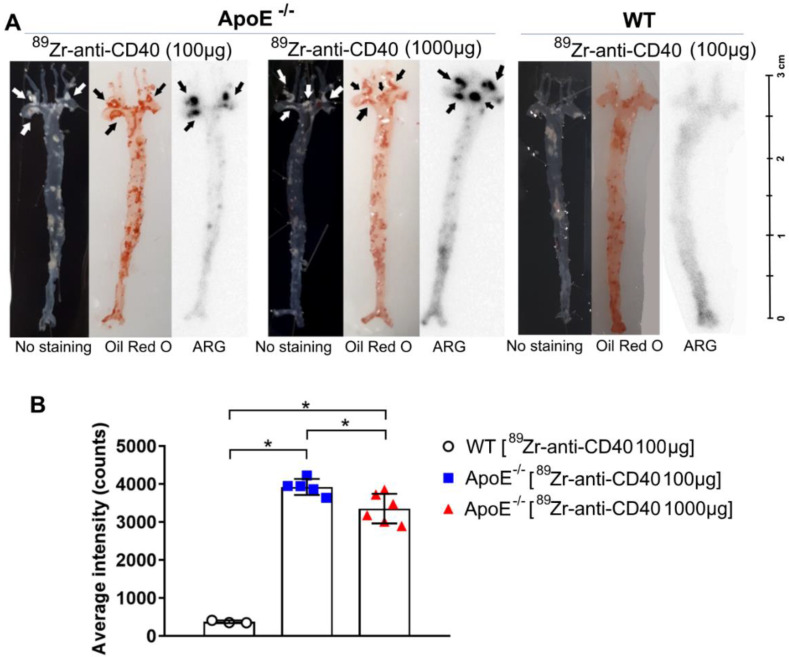
Autoradiography and Oil Red O staining of en face aortic arches demonstrate the specific binding of [^89^Zr]Zr-anti-CD40 mAb to plaque areas. (**A**) Autoradiography and Oil red O staining in both an ApoE^−/−^ and WT mouse. Arrows indicate plaque regions. (**B**) Quantification of autoradiography intensity in all groups. * *p* < 0.05; WT N = 3, ApoE^−/−^ “low dose” N = 5, ApoE^−/−^ “high dose” N = 6.

In order to validate the targeting and specific accumulation of [^89^Zr]Zr-anti-CD40 mAbs in atherosclerotic plaques in the aortic arch, ex vivo autoradiography and Oil red O staining on the aortic tissue from WT and ApoE^−/−^ mice was performed. Autoradiography images showed accumulation of the [^89^Zr]Zr-anti-CD40 mAb in atherosclerotic plaques of the aorta and the uptake correlated well with the location of plaques in these tissues as identified by Oil red O staining (Figure 5A). No plaques were observed in the WT mice (Figure 5A). Quantification of autoradiography signals showed a significantly higher uptake in the plaques of the ApoE^−/−^ “low dose” group compared to the ApoE^−/−^ “high dose” (3924 ± 211 vs. 3355 ± 389, *p* < 0.05; respectively) and WT group (375 ± 36, *p* < 0.05) (Figure 5B).

## 4. Discussion

In this study, we aimed to test the applicability of a [^89^Zr]Zr-anti-CD40 mAb in detecting atherosclerotic plaques. While the mAb remained in the circulation for a prolonged period of time, the aorta-to-blood ratio as indicated by PET/CT on days 3 and 7 post injection suggests increased binding of the [^89^Zr]Zr-anti-CD40 mAb to plaques in ApoE^−/−^ mice. WT mice without atherosclerotic lesions did not show a similar trend, indicating specificity for atherosclerotic plaques. Ex vivo biodistribution and Oil red O staining of the excised aorta in combination with autoradiography further confirmed the selective binding of the mAb to plaque tissue. CD40-rich regions, such as the lymph nodes and the spleen, served as positive control and confirmed the specific targeting of the [^89^Zr]Zr-anti-CD40 mAb.

Until now, [^18^F]FDG-PET is the gold standard to determine atherosclerotic plaque inflammation [15]. However, [^18^F]FDG also has its own distinct disadvantages. It can only detect macrophages, predominantly glycolytic ones, and can thus easily overlook other key players that contribute to a vulnerable plaque phenotype, such as activated endothelial cells and smooth muscle cells [15,16]. More selective antibodies that bind to a range of pro-inflammatory cell types could give a more precise indication of plaque vulnerability. Co-stimulatory molecules are present in a plethora of cells, including immune cells, and have been demonstrated to play a key role in the development and rupture of atherosclerotic lesions [23]. Important dyads such as CD80/86-CD28 and CD40-CD40L interactions can all contribute to a pro-inflammatory micro-milieu within the plaque, which makes them interesting imaging targets [23]. Other groups have developed immuno-PET tracers for other co-stimulatory molecules to obtain a similar advantage in plaque detection. For example, imaging CD80, another co-stimulatory checkpoint molecule present on macrophages and increasingly found in vulnerable plaques, was tested with small molecule PET tracer [^11^C]AM7 [24]. While ApoE^−/−^ mice had [^11^C]AM7 uptake in their lesions, background signals were also high. When compared to glycolytic probes such as [^18^F]FDG, a 7-fold lower uptake in standardized uptake value was found for [^11^C]AM7 in the vessel wall, thus diminishing [^11^C]AM7’s applicability to detect initial lesions with low CD80 expression [24]. Additionally, Belatacept, a CTLA4 fusion protein that binds to CD80/86, was radioactively labeled with Indium-111 and plaque uptake, was analyzed in ApoE^−/−^ mice with an arterial cuff [25]. Similar to our study, [^111^In]In-Belatacept accumulated in the lymph nodes and atherosclerotic lesions [25]. Interestingly, [^111^In]In-Belatacept uptake was also tested in human carotid specimens which showed increased in vitro binding by autoradiography, indicating a possibility for CD80/86 detection for clinical purposes [25].

CD40 was our first choice as a proof of concept for detecting vulnerable lesions as CD40 expression is not limited to (activated) macrophages, but can be found on B cells, activated endothelial cells and smooth muscle cells, and repeated experiments in our own group have demonstrated the crucial role CD40 plays in plaque development [13,14,16,26]. The results of this study suggest that the [^89^Zr]Zr-anti-CD40 mAb has successfully passed its initial preclinical validation phase for detecting CD40 atherosclerotic plaques using PET-CT imaging. As [^18^F]FDG is currently widely used, it is standard-of-care in most modern hospitals and plays a pivotal role in both the oncology and cardiovascular fields [27]. Fully replacing it with an alternative imaging strategy at this moment in time is not feasible. Nevertheless, a combination of [^18^F]FDG and [^89^Zr]Zr-anti-CD40 mAb PET-CT could be used to not only validate [^89^Zr]Zr-anti-CD40 PET scans, but to also determine whether a combination strategy leads to increased and better defined detection of vulnerable plaques. Similarly, a comparison with the previously mentioned [^111^In]In-Belatacept could validate [^89^Zr]Zr-anti-CD40 mAb as a potential imaging tool and could determine which immune checkpoint molecule is most suitable for detecting vulnerable lesions. While these comparisons could initially be performed in atherosclerotic mice, the limited size of the plaque reduces the suitability of the model. Larger animals, such as rabbits or pigs, would be better models for these more exact imaging studies as they would enable increased binding of the [^89^Zr]Zr-anti-CD40 mAb and therefore a clearer signal to detect [28,29].

Besides switching to another model organism, improvement in plaque detection could also be made by adapting the CD40 PET tracer. While mAbs have good specificity and high affinity to their corresponding targets, their long circulation time reduces their applicability in blood-rich organs [30]. Our [^89^Zr]Zr-anti-CD40 mAb circulated in the blood for an extended time period which lead to increased background in blood-rich locations, including the aorta. This complication required a prolonged waiting time before a change in aorta-to-blood ratio could be detected. Alternative options that might be able to circumvent this issue include the use of F(ab’)_2_ or Fab antibody fragments. These smaller molecules have faster blood kinetics and can be designed with similar specificity and affinity as full mAbs [31]. A recent preclinical study using a F(ab’)_2_ fragment selective for Galectin 3 was able to clearly detect binding of the [^89^Zr]Zr-DFO-Gal3-F(ab’)2 mAb to atherosclerotic lesions within 48 h post injection [32]. Furthermore, Varasteh et al. demonstrated here that Galectin 3 is a potential target to image the more anti-inflammatory M2 macrophages. Significant binding of [^89^Zr]Zr-DFO-Gal3-F(ab’)2 mAb or AlexaFluor488-coupled Gal3-F(ab’)2 was only achieved in IL4/IL10 stimulated M2 macrophages and not in M0 or M1 populations [32]. Their results are suggestive of the potential to detect different macrophage populations within the plaques using these antibody fragments in combination with a specific target. A similar probe with an antibody fragment that binds CD40 would be a potential next step to further discover the potential of CD40 as a PET/CT marker in atherosclerotic aortas, especially considering the improved results observed in the “low dose” group compared to the “high dose” group in this study.

As this CD40 antibody is not cross-reactive with human CD40, we were unable to use our [^89^Zr]Zr-anti-CD40 on human samples. Nevertheless, validation of a similar, but human, CD40 binding tracer in patient samples could clarify its potential. A possible next step could be to label Iscalimab, a human anti-human CD40 monoclonal antibody produced by Novartis, which is currently being tested in SLE, Sjögren’s disease and Type 1 Diabetes Mellitus (Clinical trial # NCT03656562, NCT03905525, NCT04129528) [33]. Nevertheless, before these advances can be made, further validation experiments have to be executed. For now, we have shown that PET imaging targeting CD40 is a promising target to detect vulnerable plaques as increased uptake is detected in the aorta. The full potential of [^89^Zr]Zr-anti-CD40 mAb remains to be elucidated, but future experiments will verify the current findings and could contribute to the further validation of the tracer as an imaging tool to detect vulnerable lesions. Finally, the question remains whether CD40 could also be a therapeutic target, so that imaging can be performed in a theranostic setting, which would increase the demand for further validation even more.

## 5. Conclusions

In conclusion, this proof of concept study showed that PET/CT imaging with [^89^Zr]Zr-anti-CD40 mAb can detect atherosclerotic plaques. As CD40 is associated with plaque vulnerability, [^89^Zr]Zr-anti-CD40 mAb has the potential to become a valuable tracer for detection of vulnerable atherosclerotic plaques.

## Data Availability

Not applicable.

## Supplementary Materials

The following are available online at https://www.mdpi.com/article/10.3390/biology11030408/s1. Figure S1. Representative examples of regions of interest (ROIs) drawn on their corresponding organ. (A) ROIs drawn on liver (purple), spleen (red), lymph node (green) and muscle (yellow). (B) ROIs drawn on aorta arch (red) and left ventricle (blue). Figure S2. SE-HPLC chromatogram of formulated product [^89^Zr]Zr-anti-CD40mAb for injection into WT animals. Concentration of anti-CD40 mAb is 0.69 mg/mL. Top panel: radioactivity, bottom panel: UV at 280 nm. Figure S3. SE-HPLC chromatogram of formulated product [^89^Zr]Zr-anti-CD40mAb for injection into for ApoE^−/−^ animals. Concentration of anti-CD40 mAb is 0.69 mg/mL. Top panel: radioactivity, bottom panel: UV at 280 nm. Figure S4. SE-HPLC chromatogram of formulated product [^89^Zr]Zr-anti-CD40mAb + cold antibody for injection into for ApoE^−/−^ animals “High dose”. Concentration of anti-CD40 mAb is 4.95 mg/mL. Top panel: radioactivity, bottom panel: UV at 280 nm. Table S1. PET imaging quantifications of [^89^Zr]Zr-anti-CD40 mAb at day 3 p.i. in WT and ApoE^−/−^ mice. Table S2. PET imaging quantifications of [^89^Zr]Zr-anti-CD40 mAb at day 7 p.i. in WT and ApoE^−/−^ mice. Table S3. Ex vivo biodistribution of [^89^Zr]Zr-anti-CD40 mAb at day 7 p.i. in WT and ApoE^−/−^ mice. Table S4. Blood kinetics of [^89^Zr]Zr-anti-CD40 mAb at day 1, 2, 3 and 7 p.i. in WT and ApoE^−/−^ mice.
