# Supplementary material for: Immuno-PET Imaging of Atherosclerotic Plaques with [89Zr]Zr-Anti-CD40 mAb—Proof of Concept
Source: Biology (Basel). 2022 Mar 6;11(3):408. doi: 10.3390/biology11030408 (PMC8944956; doi:10.3390/biology11030408)
Supplement: Supplementary file 1 [file biology-11-00408-s001.zip › biology-1615120-SI.pdf]

Supplementary Materials

1 Supplementary Figures and tables

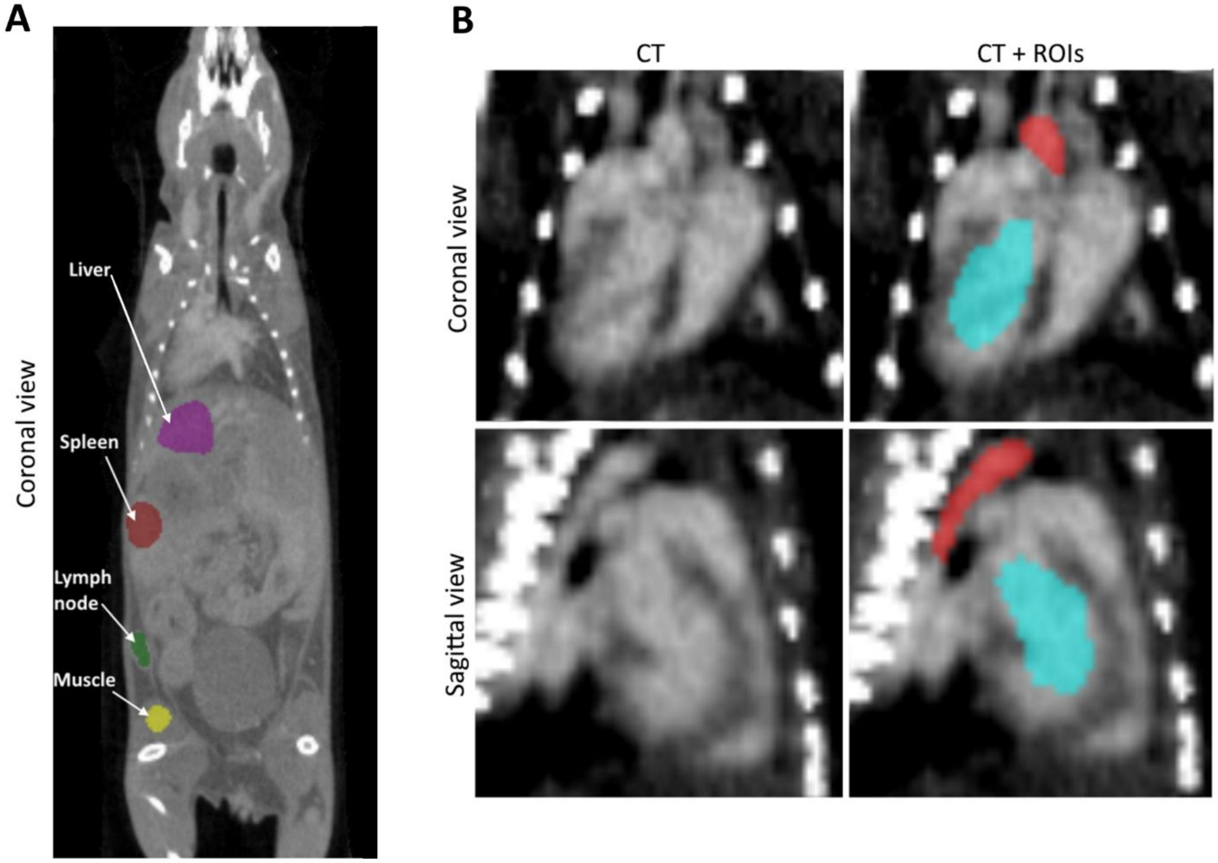

Figure S1. Representative examples of regions of interest (ROIs) drawn on their corresponding organ. **(A)** ROIs drawn on liver (purple), spleen (red), lymph node (green) and muscle (yellow). **(B)** ROIs drawn on aorta arch (red) and left ventricle (blue).

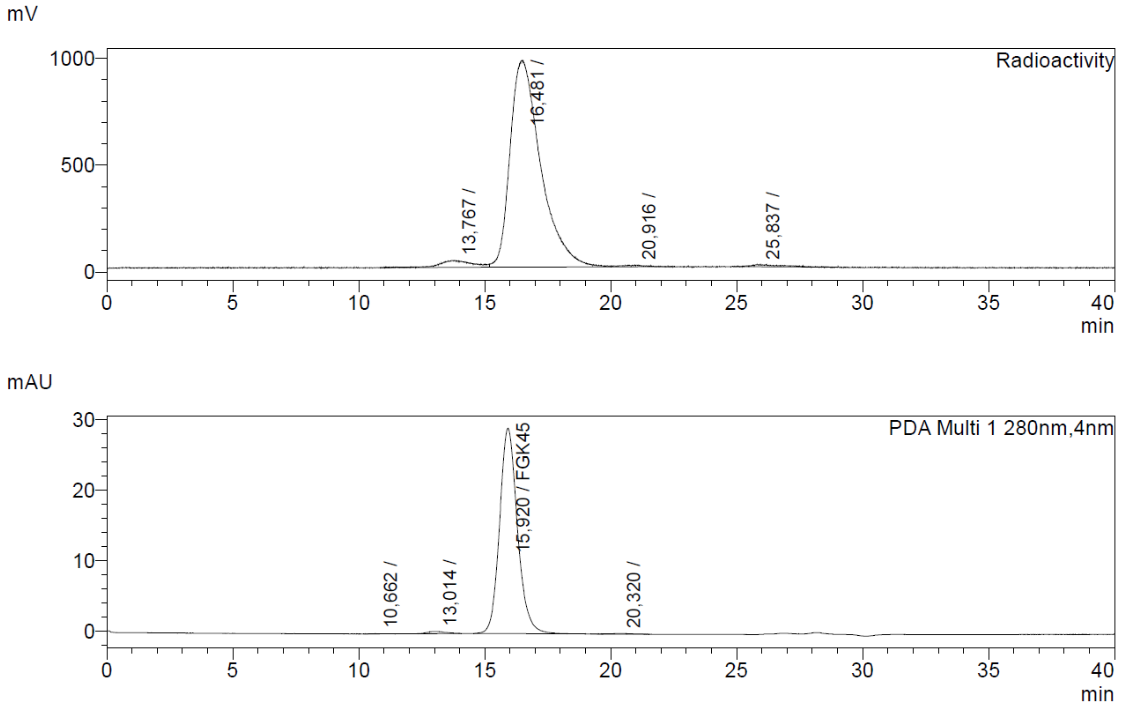

Figure S2. SE-HPLC chromatogram of formulated product [^{89}Zr]Zr-anti-CD40mAb for injection into WT animals. Concentration of anti-CD40 mAb is 0.69 mg/mL. Top panel: radioactivity, bottom panel: UV at 280 nm.

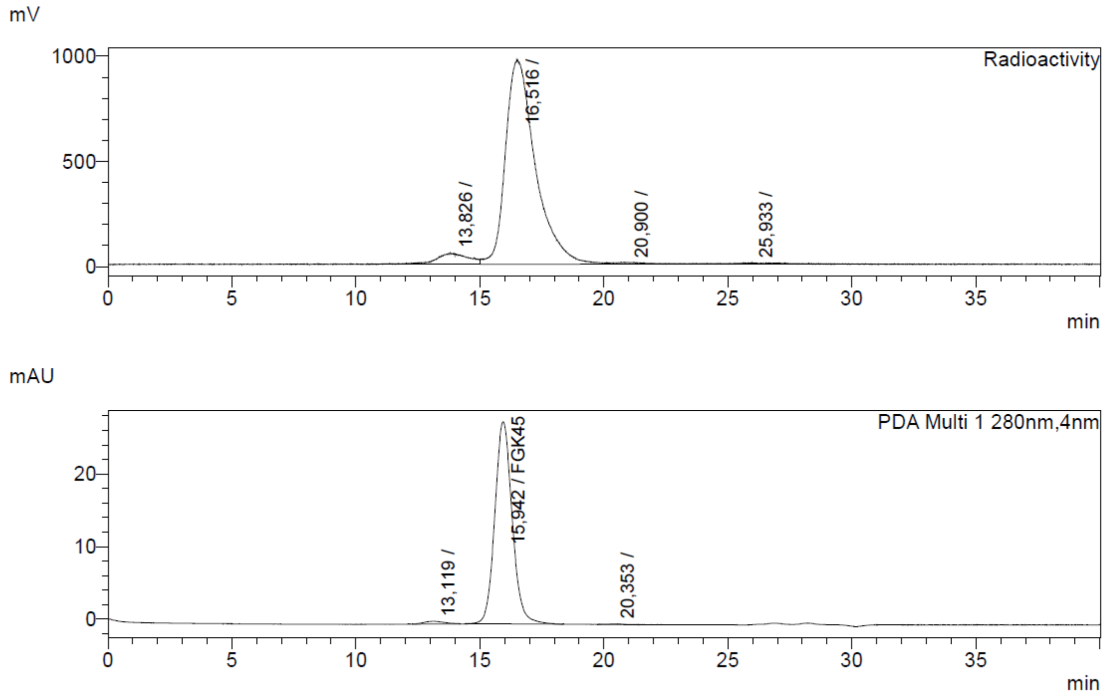

Figure S3. SE-HPLC chromatogram of formulated product [^{89}Zr]Zr-anti-CD40mAb for injection into for ApoE$^{-/-}$ animals. Concentration of anti-CD40 mAb is 0.69 mg/mL. Top panel: radioactivity, bottom panel: UV at 280 nm.

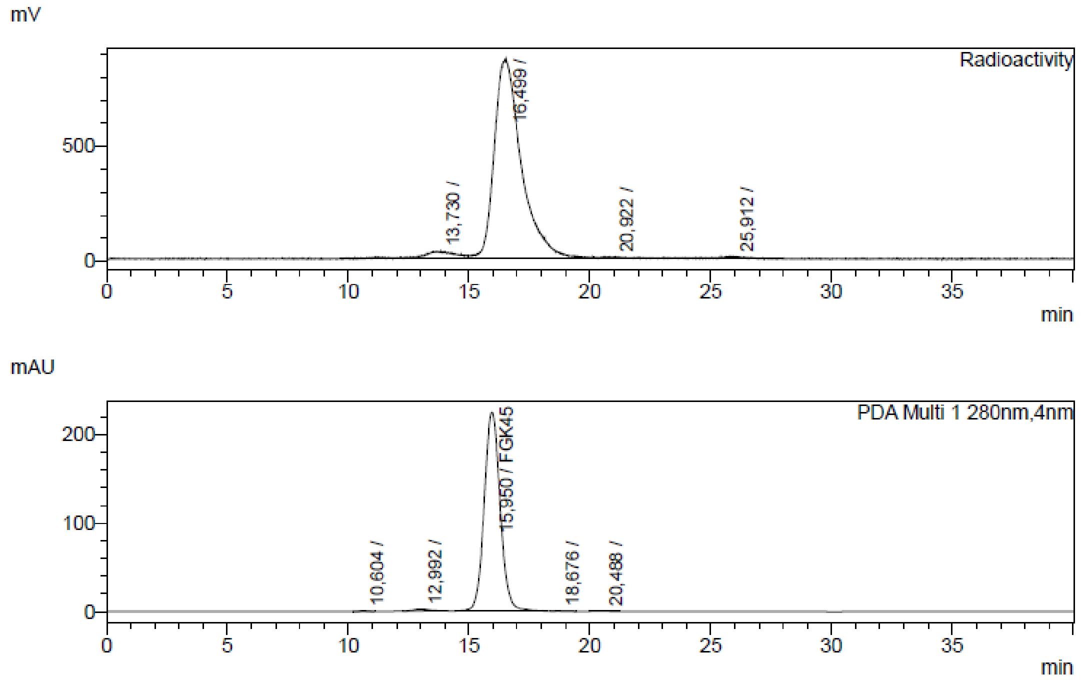

Figure S4. SE-HPLC chromatogram of formulated product [^{89}Zr]Zr-anti-CD40mAb + cold antibody for injection into for ApoE$^{-/-}$ animals "High dose". Concentration of anti-CD40 mAb is 4.95 mg/mL. Top panel: radioactivity, bottom panel: UV at 280 nm.

Table S1: PET imaging quantifications of [89Zr]Zr-anti-CD40 mAb at day 3 p.i. in WT and ApoE−/− mice.

	**WT ([^{89}Zr]Zr-anti-CD40 100 µg)**	**ApoE$^{-/-}$ ([^{89}Zr]Zr -anti-CD40 100 µg)**	**ApoE$^{-/-}$ ([^{89}Zr]Zr -anti-CD40 1000 µg)**
Liver	9.96 ± 1.24	10.34 ± 1.81	10.19 ± 1.00
Spleen	18.39 ± 2.13	15.14 ± 1.66	9.96 ± 1.03
Lymph nodes	7.80 ± 1.42	7.39 ± 1.03	3.81 ± 0.67
Muscle	0.65 ± 0.18	0.62 ± 0.29	0.95 ± 0.27
Aorta	4.74 ± 1.48	3.70 ±0.50	6.73 ± 0.65
Blood	4.18 ± 1.58	2.75 ± 0.40	6.70 ± 1.35
Aorta/ blood ratio	1.16 ± 0.08	1.37 ± 0.03	1.08 ± 0.07

Data is presented in percentage injected dose/gram tissue (%ID/g). Average ± SD.

Table S2: PET imaging quantifications of [^{89}Zr]Zr-anti-CD40 mAb at day 7 p.i. in WT and ApoE$^{-/-}$ mice**.**

	**WT ([^{89}Zr]Zr -anti-CD40 100 µg)**	**ApoE$^{-/-}$ ([^{89}Zr]Zr -anti-CD40 100 µg)**	**ApoE$^{-/-}$ ([^{89}Zr]Zr -anti-CD40 1000 µg)**
Liver	8.67 ± 0.68	6.64 ± 1.43	7.89 ± 1.27
Spleen	14.02 ± 2.03	10.41 ± 1.38	6.56 ± 1.06
Lymph nodes	4.94 ± 1.36	3.88 ± 0.97	3.36 ± 0.83
Muscle	0.20 ± 0.07	0.10 ± 0.03	0.25 ± 0.06
Aorta	0.89 ± 0.20	0.71 ± 0.13	1.41 ± 0.56
Blood	0.48 ± 0.24	0.21 ± 0.02	0.74 ± 0.40
Aorta/ blood ratio	1.72 ± 0.30	3.34 ± 0.62	2.05 ± 0.47

Data is presented in percentage injected dose/gram tissue (%ID/g). Average ± SD.

Table S3: *Ex vivo* biodistribution of [^{89}Zr]Zr-anti-CD40 mAb at day 7 p.i. in WT and ApoE$^{-/-}$ mice.

	**WT ([^{89}Zr]Zr -anti-CD40 100 µg)**	**ApoE$^{-/-}$ ([^{89}Zr]Zr -anti-CD40 100 µg)**	**ApoE$^{-/-}$ ([^{89}Zr]Zr -anti-CD40 1000 µg)**
Blood	0.43 ± 0.39	0.15 ± 0.04	0.92 ± 0.38
Heart	0.52 ± 0.14	0.21 ± 0.03	0.49 ± 0.11
Liver	10.63 ± 0.96	8.17 ± 2.17	8.67 ± 1.93
Spleen	23.89 ± 4.75	15.39 ± 3.26	9.92 ± 1.51
Kidney	3.07 ± 0.23	2.08 ± 0.37	2.63 ± 0.96
Muscle	0.15 ± 0.04	0.08 ± 0.01	0.26 ± 0.09
Femur	2.10 ± 0.40	1.34 ± 0.39	1.72 ± 0.16
Lymph nodes	12.23 ± 4.08	7.62 ± 2.51	5.66 ± 1.43
Thymus	4.33 ± 0.86	3.77 ± 0.91	4.44 ± 1.11

Data is presented in percentage injected dose/gram tissue (%ID/g). Average ± SD.

Table S4: Blood kinetics of [^{89}Zr]Zr-anti-CD40 mAb at day 1, 2, 3 and 7 p.i. in WT and ApoE$^{-/-}$ mice**.**

Post injection	**WT ([^{89}Zr]Zr - anti-CD40 100 µg)**	**ApoE$^{-/-}$ ([^{89}Zr]Zr -anti-CD40 100 µg)**	**ApoE$^{-/-}$ ([^{89}Zr]Zr - anti-CD40 1000 µg)**
Day 1	13.47 ± 1.45	14.09 ± 1.52	17.43 ± 2.12
Day 2	7.78 ± 1.20	8.02 ± 1.68	12.99 ± 1.42
Day 3	4.90 ± 1.62	3.90 ± 0.95	7.99 ± 1.88
Day 7	0.43 ± 0.39	0.15 ± 0.04	0.92 ± 0.38

Data is presented in percentage injected dose/gram tissue (%ID/g). Average ± SD.
